# Supplementary material for: Role and mechanism of NCAPD3 in promoting malignant behaviors in gastric cancer
Source: Front Pharmacol. 2024 Apr 22;15:1341039. doi: 10.3389/fphar.2024.1341039 (PMC11070777; doi:10.3389/fphar.2024.1341039)
Supplement: Supplementary file 11 [file DataSheet2.ZIP › GSEA/Transcipt factor/my_analysis.Gsea.1599461814734/ZSCAN5DP_TARGET_GENES.html]

Details for gene set ZSCAN5DP\_TARGET\_GENES[GSEA]

|  || Dataset | filtered\_dataset.sample\_info.cls#WT\_versus\_NCAPD3\_MUT |
| Phenotype | sample\_info.cls#WT\_versus\_NCAPD3\_MUT |
| Upregulated in class | WT |
| GeneSet | ZSCAN5DP\_TARGET\_GENES |
| Enrichment Score (ES) | 0.21543682 |
| Normalized Enrichment Score (NES) | 1.6514659 |
| Nominal p-value | 0.009985735 |
| FDR q-value | 0.5451133 |
| FWER p-Value | 1.0 |
Table: GSEA Results Summary

  

Fig 1: Enrichment plot: ZSCAN5DP\_TARGET\_GENES      
 Profile of the Running ES Score & Positions of GeneSet Members on the Rank Ordered List

  

| SYMBOL | TITLE | RANK IN GENE LIST | RANK METRIC SCORE | RUNNING ES | CORE ENRICHMENT || 1 | 2935 | GSPT1 | 5 | 1.156 | 0.0095 | Yes |
| 2 | 5514 | PPP1R10 | 11 | 1.087 | 0.0183 | Yes |
| 3 | 7571 | ZNF23 | 25 | 1.018 | 0.0198 | Yes |
| 4 | 23253 | ANKRD12 | 34 | 0.977 | 0.0248 | Yes |
| 5 | 55727 | BTBD7 | 38 | 0.954 | 0.0336 | Yes |
| 6 | 9759 | HDAC4 | 46 | 0.925 | 0.0388 | Yes |
| 7 | 284942 | RPL23AP82 | 48 | 0.923 | 0.0488 | Yes |
| 8 | 376940 | ZC3H6 | 50 | 0.914 | 0.0587 | Yes |
| 9 | 1497 | CTNS | 58 | 0.883 | 0.0635 | Yes |
| 10 | 57542 | KLHL42 | 59 | 0.876 | 0.0737 | Yes |
| 11 | 23322 | RPGRIP1L | 60 | 0.871 | 0.0839 | Yes |
| 12 | 2120 | ETV6 | 63 | 0.862 | 0.0924 | Yes |
| 13 | 23054 | NCOA6 | 77 | 0.838 | 0.0918 | Yes |
| 14 | 79020 | C7orf25 | 97 | 0.793 | 0.0859 | Yes |
| 15 | 55006 | TRMT61B | 101 | 0.782 | 0.0926 | Yes |
| 16 | 50861 | STMN3 | 126 | 0.777 | 0.0826 | Yes |
| 17 | 51643 | TMBIM4 | 129 | 0.773 | 0.0900 | Yes |
| 18 | 4193 | MDM2 | 131 | 0.769 | 0.0982 | Yes |
| 19 | 7248 | TSC1 | 158 | 0.718 | 0.0858 | Yes |
| 20 | 2186 | BPTF | 159 | 0.717 | 0.0942 | Yes |
| 21 | 10795 | ZNF268 | 168 | 0.710 | 0.0961 | Yes |
| 22 | 29940 | DSE | 174 | 0.703 | 0.1004 | Yes |
| 23 | 23450 | SF3B3 | 178 | 0.697 | 0.1061 | Yes |
| 24 | 51018 | RRP15 | 192 | 0.685 | 0.1038 | Yes |
| 25 | 84190 | METTL25 | 195 | 0.684 | 0.1102 | Yes |
| 26 | 9897 | KIAA0196 | 197 | 0.682 | 0.1173 | Yes |
| 27 | 145389 | SLC38A6 | 202 | 0.673 | 0.1220 | Yes |
| 28 | 57521 | RPTOR | 239 | 0.645 | 0.1008 | Yes |
| 29 | 161779 | PGBD4 | 242 | 0.637 | 0.1066 | Yes |
| 30 | 64794 | DDX31 | 248 | 0.633 | 0.1101 | Yes |
| 31 | 55238 | SLC38A7 | 250 | 0.630 | 0.1166 | Yes |
| 32 | 528 | ATP6V1C1 | 254 | 0.625 | 0.1215 | Yes |
| 33 | 22845 | DOLK | 269 | 0.618 | 0.1176 | Yes |
| 34 | 4238 | MFAP3 | 279 | 0.609 | 0.1175 | Yes |
| 35 | 80124 | VCPIP1 | 282 | 0.607 | 0.1230 | Yes |
| 36 | 84874 | ZNF514 | 283 | 0.606 | 0.1301 | Yes |
| 37 | 23036 | ZNF292 | 288 | 0.602 | 0.1340 | Yes |
| 38 | 81889 | FAHD1 | 299 | 0.592 | 0.1329 | Yes |
| 39 | 25852 | ARMC8 | 307 | 0.588 | 0.1342 | Yes |
| 40 | 79443 | FYCO1 | 313 | 0.586 | 0.1370 | Yes |
| 41 | 6715 | SRD5A1 | 325 | 0.578 | 0.1350 | Yes |
| 42 | 8125 | ANP32A | 326 | 0.578 | 0.1418 | Yes |
| 43 | 84135 | UTP15 | 330 | 0.576 | 0.1461 | Yes |
| 44 | 94107 | TMEM203 | 333 | 0.574 | 0.1512 | Yes |
| 45 | 56910 | STARD7 | 335 | 0.573 | 0.1571 | Yes |
| 46 | 4297 | KMT2A | 337 | 0.573 | 0.1631 | Yes |
| 47 | 92399 | MRRF | 347 | 0.569 | 0.1625 | Yes |
| 48 | 2965 | GTF2H1 | 356 | 0.563 | 0.1627 | Yes |
| 49 | 5718 | PSMD12 | 372 | 0.552 | 0.1572 | Yes |
| 50 | 64327 | LMBR1 | 378 | 0.547 | 0.1596 | Yes |
| 51 | 5814 | PURB | 399 | 0.536 | 0.1499 | Yes |
| 52 | 60526 | LDAH | 403 | 0.535 | 0.1537 | Yes |
| 53 | 23031 | MAST3 | 406 | 0.534 | 0.1584 | Yes |
| 54 | 147007 | TMEM199 | 410 | 0.533 | 0.1622 | Yes |
| 55 | 63929 | XPNPEP3 | 416 | 0.526 | 0.1644 | Yes |
| 56 | 84274 | COQ5 | 419 | 0.523 | 0.1689 | Yes |
| 57 | 805 | CALM2 | 423 | 0.518 | 0.1726 | Yes |
| 58 | 801 | CALM1 | 424 | 0.518 | 0.1786 | Yes |
| 59 | 4771 | NF2 | 428 | 0.516 | 0.1823 | Yes |
| 60 | 6256 | RXRA | 439 | 0.509 | 0.1802 | Yes |
| 61 | 22796 | COG2 | 445 | 0.507 | 0.1822 | Yes |
| 62 | 57478 | USP31 | 448 | 0.506 | 0.1865 | Yes |
| 63 | 23192 | ATG4B | 452 | 0.505 | 0.1900 | Yes |
| 64 | 5510 | PPP1R7 | 490 | 0.478 | 0.1660 | Yes |
| 65 | 29101 | SSU72 | 496 | 0.475 | 0.1676 | Yes |
| 66 | 11060 | WWP2 | 503 | 0.471 | 0.1683 | Yes |
| 67 | 3184 | HNRNPD | 508 | 0.470 | 0.1706 | Yes |
| 68 | 54920 | DUS2 | 509 | 0.470 | 0.1761 | Yes |
| 69 | 5188 | GATB | 512 | 0.469 | 0.1800 | Yes |
| 70 | 104 | ADARB1 | 518 | 0.463 | 0.1814 | Yes |
| 71 | 1200 | TPP1 | 536 | 0.454 | 0.1731 | Yes |
| 72 | 29090 | TIMM21 | 537 | 0.453 | 0.1784 | Yes |
| 73 | 55527 | FEM1A | 543 | 0.449 | 0.1797 | Yes |
| 74 | 3609 | ILF3 | 544 | 0.449 | 0.1850 | Yes |
| 75 | 1819 | DRG2 | 550 | 0.447 | 0.1862 | Yes |
| 76 | 9416 | DDX23 | 553 | 0.445 | 0.1898 | Yes |
| 77 | 84951 | TNS4 | 554 | 0.445 | 0.1950 | Yes |
| 78 | 79080 | CCDC86 | 555 | 0.443 | 0.2002 | Yes |
| 79 | 6632 | SNRPD1 | 563 | 0.440 | 0.1998 | Yes |
| 80 | 23313 | KIAA0930 | 587 | 0.433 | 0.1864 | Yes |
| 81 | 26057 | ANKRD17 | 589 | 0.431 | 0.1907 | Yes |
| 82 | 23195 | MDN1 | 596 | 0.426 | 0.1909 | Yes |
| 83 | 54951 | COMMD8 | 600 | 0.423 | 0.1934 | Yes |
| 84 | 8021 | NUP214 | 611 | 0.417 | 0.1903 | Yes |
| 85 | 22847 | ZNF507 | 618 | 0.413 | 0.1904 | Yes |
| 86 | 54663 | WDR74 | 628 | 0.408 | 0.1879 | Yes |
| 87 | 23274 | CLEC16A | 629 | 0.408 | 0.1927 | Yes |
| 88 | 26953 | RANBP6 | 630 | 0.406 | 0.1975 | Yes |
| 89 | 10975 | UQCR11 | 632 | 0.406 | 0.2014 | Yes |
| 90 | 79807 | GSTCD | 644 | 0.402 | 0.1973 | Yes |
| 91 | 22929 | SEPHS1 | 645 | 0.401 | 0.2020 | Yes |
| 92 | 29890 | RBM15B | 651 | 0.400 | 0.2027 | Yes |
| 93 | 11159 | RABL2A | 653 | 0.399 | 0.2066 | Yes |
| 94 | 11158 | RABL2B | 654 | 0.399 | 0.2112 | Yes |
| 95 | 26747 | NUFIP1 | 666 | 0.394 | 0.2071 | Yes |
| 96 | 51603 | METTL13 | 669 | 0.391 | 0.2100 | Yes |
| 97 | 9453 | GGPS1 | 677 | 0.389 | 0.2090 | Yes |
| 98 | 132949 | AASDH | 689 | 0.384 | 0.2047 | Yes |
| 99 | 10452 | TOMM40 | 696 | 0.380 | 0.2043 | Yes |
| 100 | 10607 | TBL3 | 698 | 0.380 | 0.2080 | Yes |
| 101 | 84975 | MFSD5 | 702 | 0.376 | 0.2100 | Yes |
| 102 | 6919 | TCEA2 | 712 | 0.368 | 0.2071 | Yes |
| 103 | 23350 | U2SURP | 719 | 0.364 | 0.2066 | Yes |
| 104 | 55122 | AKIRIN2 | 721 | 0.363 | 0.2100 | Yes |
| 105 | 122809 | SOCS4 | 726 | 0.360 | 0.2110 | Yes |
| 106 | 153562 | MARVELD2 | 732 | 0.359 | 0.2112 | Yes |
| 107 | 134430 | WDR36 | 733 | 0.359 | 0.2154 | Yes |
| 108 | 58509 | CACTIN | 748 | 0.351 | 0.2084 | No |
| 109 | 6605 | SMARCE1 | 749 | 0.349 | 0.2124 | No |
| 110 | 55178 | RNMTL1 | 771 | 0.337 | 0.1996 | No |
| 111 | 54939 | COMMD4 | 777 | 0.335 | 0.1995 | No |
| 112 | 4670 | HNRNPM | 790 | 0.330 | 0.1938 | No |
| 113 | 51154 | MRTO4 | 794 | 0.327 | 0.1952 | No |
| 114 | 9749 | PHACTR2 | 806 | 0.319 | 0.1902 | No |
| 115 | 51575 | ESF1 | 812 | 0.309 | 0.1898 | No |
| 116 | 4285 | MIPEP | 815 | 0.307 | 0.1918 | No |
| 117 | 9343 | EFTUD2 | 818 | 0.305 | 0.1937 | No |
| 118 | 11091 | WDR5 | 822 | 0.304 | 0.1949 | No |
| 119 | 26121 | PRPF31 | 825 | 0.301 | 0.1968 | No |
| 120 | 387680 | FAM21A | 828 | 0.297 | 0.1987 | No |
| 121 | 253725 | FAM21C | 829 | 0.297 | 0.2022 | No |
| 122 | 6732 | SRPK1 | 835 | 0.291 | 0.2016 | No |
| 123 | 8835 | SOCS2 | 840 | 0.275 | 0.2016 | No |
| 124 | 6515 | SLC2A3 | 873 | -0.283 | 0.1793 | No |
| 125 | 51278 | IER5 | 874 | -0.283 | 0.1827 | No |
| 126 | 3005 | H1F0 | 880 | -0.294 | 0.1821 | No |
| 127 | 23412 | COMMD3 | 894 | -0.312 | 0.1754 | No |
| 128 | 55652 | SLC48A1 | 897 | -0.317 | 0.1775 | No |
| 129 | 4616 | GADD45B | 904 | -0.330 | 0.1765 | No |
| 130 | 4088 | SMAD3 | 930 | -0.353 | 0.1607 | No |
| 131 | 901 | CCNG2 | 998 | -0.405 | 0.1119 | No |
| 132 | 51646 | YPEL5 | 1014 | -0.413 | 0.1047 | No |
| 133 | 53349 | ZFYVE1 | 1018 | -0.417 | 0.1072 | No |
| 134 | 121268 | RHEBL1 | 1028 | -0.428 | 0.1050 | No |
| 135 | 1628 | DBP | 1029 | -0.429 | 0.1101 | No |
| 136 | 54676 | GTPBP2 | 1050 | -0.447 | 0.0993 | No |
| 137 | 81502 | HM13 | 1057 | -0.450 | 0.0998 | No |
| 138 | 81631 | MAP1LC3B | 1060 | -0.452 | 0.1035 | No |
| 139 | 1308 | COL17A1 | 1065 | -0.455 | 0.1056 | No |
| 140 | 8609 | KLF7 | 1071 | -0.457 | 0.1070 | No |
| 141 | 283 | ANG | 1074 | -0.458 | 0.1107 | No |
| 142 | 7168 | TPM1 | 1086 | -0.465 | 0.1074 | No |
| 143 | 11221 | DUSP10 | 1095 | -0.475 | 0.1065 | No |
| 144 | 2729 | GCLC | 1123 | -0.497 | 0.0908 | No |
| 145 | 146057 | TTBK2 | 1148 | -0.513 | 0.0776 | No |
| 146 | 84925 | DIRC2 | 1153 | -0.516 | 0.0804 | No |
| 147 | 92014 | SLC25A51 | 1155 | -0.519 | 0.0857 | No |
| 148 | 1540 | CYLD | 1162 | -0.530 | 0.0871 | No |
| 149 | 665 | BNIP3L | 1185 | -0.556 | 0.0760 | No |
| 150 | 153222 | CREBRF | 1187 | -0.559 | 0.0818 | No |
| 151 | 6038 | RNASE4 | 1194 | -0.570 | 0.0836 | No |
| 152 | 57649 | PHF12 | 1212 | -0.588 | 0.0769 | No |
| 153 | 56995 | TULP4 | 1216 | -0.591 | 0.0815 | No |
| 154 | 145508 | CEP128 | 1236 | -0.614 | 0.0734 | No |
| 155 | 8412 | BCAR3 | 1261 | -0.648 | 0.0618 | No |
| 156 | 9262 | STK17B | 1268 | -0.659 | 0.0648 | No |
| 157 | 7994 | KAT6A | 1279 | -0.666 | 0.0646 | No |
| 158 | 57561 | ARRDC3 | 1324 | -0.744 | 0.0381 | No |
| 159 | 2591 | GALNT3 | 1403 | -1.195 | -0.0103 | No |
| 160 | 389668 | XKR9 | 1405 | -1.286 | 0.0040 | No |
Table: GSEA details [plain text format]

  

Fig 2: ZSCAN5DP\_TARGET\_GENES      
 Blue-Pink O' Gram in the Space of the Analyzed GeneSet

  

Fig 3: ZSCAN5DP\_TARGET\_GENES: Random ES distribution      
 Gene set null distribution of ES for **ZSCAN5DP\_TARGET\_GENES**
